# A Comprehensive Review of the Literature: Does an Optimal Type of Anastomosis Exist for One Anastomosis Gastric Bypass?

**DOI:** 10.7759/cureus.71065

**Published:** 2024-10-08

**Authors:** Amir Farah, Anna Tatakis, Mohammad Safadi, Sa'd Sayida

**Affiliations:** 1 General Surgery, Medical College of Wisconsin, Milwaukee, USA; 2 General Surgery, EMMS Nazareth Hospital, Nazareth, ISR

**Keywords:** anastomosis, bariatric, circular stapled anastomosis, gastrojejunostomy, hand sewn anastomosis, linear stapled anastomosis, mini gastric bypass, obesity, one anastomosis gastric bypass, weight loss

## Abstract

One anastomosis gastric bypass (OAGB) is a popular bariatric procedure known for its efficacy in promoting weight loss and improving metabolic outcomes. However, the optimal anastomotic technique for OAGB remains a subject of debate. This literature review comprehensively examines the three primary anastomotic techniques - linear stapled, circular stapled, and hand-sewn - to determine their suitability for OAGB.

Linear stapled anastomosis is favored for its shorter operative time and lower complication rates, such as a reduced incidence of gastrojejunal stenosis. However, its larger anastomotic diameter increases the risk of marginal ulcers due to greater exposure of the gastric mucosa to bile and gastric acids. Circular stapled anastomosis offers a uniform and consistent lumen, which may reduce the risk of postoperative stenosis but is associated with a higher incidence of strictures and ulcers, making it less ideal for use in OAGB. Hand-sewn anastomosis, while time-intensive, provides superior control over anastomotic size and tension, resulting in the lowest rates of strictures and anastomotic leaks, although its effectiveness is highly dependent on surgical expertise.

Overall, the current literature lacks large-scale, multicenter studies directly comparing these techniques in the context of OAGB. Future research should focus on randomized controlled trials to establish the most effective and safe anastomotic method for this procedure. Understanding the nuanced benefits and drawbacks of each technique is crucial for optimizing clinical outcomes in OAGB.

## Introduction and background

Obesity is a significant global health challenge, affecting over 650 million adults worldwide [1,2]. It contributes substantially to health complications and places a considerable burden on healthcare systems due to the high costs associated with managing related conditions [1,2]. Effective long-term treatment strategies, such as bariatric surgery, are essential to combat the growing epidemic of obesity, as highlighted by its substantial impact on health systems and the global burden of disease [1,2].

Bariatric surgery in general, and one anastomosis gastric bypass (OAGB) in particular, is recognized as one of the most effective interventions for achieving sustained weight loss and improving obesity-related health issues, especially when conservative measures such as diet and exercise have failed [3]. OAGB has gained popularity due to its simplicity, efficacy, and relatively low complication rates compared to procedures like Roux-en-Y gastric bypass (RYGB) and sleeve gastrectomy (SG) [4,5]. The technique of OAGB involves creating a narrow gastric pouch anastomosed to a loop of the jejunum, combining both restrictive and malabsorptive elements to aid in weight loss and metabolic improvement [6]. OAGB has been proposed as a simpler and potentially more effective alternative to RYGB for the treatment of obesity and type 2 diabetes, due to its single anastomosis and lower complication rates [7]. Studies have also demonstrated that OAGB not only provides significant weight loss but also results in superior glycemic control and improvement in insulin sensitivity when compared to other bariatric procedures [8,9]. This highlights its potential as a preferred surgical option for patients with obesity and diabetes.

This review will explore the three primary anastomotic techniques used in OAGB - linear stapled, circular stapled, and hand-sewn - discussing their technical details, benefits, and limitations. A comprehensive comparative analysis will follow to highlight the strengths and weaknesses of each method and their implications for clinical practice, with a specific focus on their potential application in OAGB. 

## Review

Linear stapled anastomosis

Linear stapled anastomosis involves using a linear cutting stapler to create a side-to-side or end-to-side anastomosis between the gastric pouch and the jejunal limb. This technique is widely utilized due to its operational simplicity, shorter operative time, and relatively low complication rates.

Technical Aspects

In a typical linear stapled anastomosis, a small opening is made in both the gastric pouch and the jejunum. A linear stapler is then introduced and fired to create an anastomosis, and the common enterotomy is closed using one or two layers of sutures [10]. The resulting anastomosis is tension-free, allowing for good gastric emptying and minimizing the risk of strictures [10].

Operative Efficiency

Linear stapled anastomosis is generally favored for its operational efficiency. The average operative time is reported to be around 92.5 minutes, which is significantly shorter compared to other techniques [11]. This reduction in operative time is particularly advantageous in high-volume centers, where minimizing surgical duration is essential to optimize resource utilization and reduce the risk of anesthesia-related complications.

The shorter operative time is attributed to the relative simplicity of the linear stapled technique, which involves fewer steps than the circular stapled or hand-sewn methods. For example, the linear stapler allows for a quick and efficient creation of the anastomosis, and the closure of the common enterotomy can be completed in a single layer using a continuous suture, further reducing the time required [11].

Complication Rates

Studies have shown that linear stapled anastomosis results in fewer complications compared to other techniques. Netto et al. [11] found a significantly lower incidence of gastrojejunal stenosis (0.5%) compared to circular stapled (1.7%) and hand-sewn techniques (3%). Similarly, Major et al. observed a 1% stenosis rate, further supporting the effectiveness of linear stapling in reducing the risk of strictures [12].

However, the larger anastomotic diameter created by linear staplers (1.5 cm vs. 0.98 cm for circular staplers) can increase the risk of marginal ulcers [13]. Owens and Sczepaniak reported a marginal ulcer rate of 7.5% in patients with larger anastomotic diameters, compared to 3.2% in those with smaller diameters [13]. This suggests that while linear stapled anastomosis can enhance postoperative outcomes, it may also lead to complications such as increased exposure of the gastric mucosa to bile and gastric acids.

Application in OAGB

While linear stapled anastomosis is commonly used in bariatric surgeries like RYGB, its application in OAGB has not been extensively studied. The main concern with using a larger anastomotic diameter in OAGB is the potential increase in bile reflux, which could lead to marginal ulcers and esophagitis [11]. Studies focusing on OAGB are needed to establish the optimal anastomotic size and technique to minimize these complications.

Circular stapled anastomosis

Circular stapled anastomosis involves placing a circular stapler’s anvil into the gastric pouch and connecting it through a small enterotomy in the jejunal limb. When fired, the stapler creates a uniform, ring-shaped anastomosis that provides consistent lumen diameter.

Technical Aspects

This technique begins by placing the circular stapler’s anvil into the gastric pouch through an opening in the proximal stomach. The circular stapler is then connected through a small enterotomy in the jejunal limb, and when fired, it creates a ring-shaped anastomosis [14]. The integrity of the anastomosis is typically checked intraoperatively using endoscopy to ensure no leaks are present [14].

Operative Efficiency

Circular stapled anastomosis tends to have longer operative times compared to linear stapling. Studies report an average operative time of 110.6 minutes for circular stapling, which is significantly longer than the time required for linear stapling [11]. This increased time can be attributed to the more complex steps involved in placing the circular stapler and ensuring precise alignment of the anvil and the stapler body.

One of the primary reasons for the extended operative time is the need for accurate positioning of the stapler's anvil within the gastric pouch and precise alignment with the jejunal limb. This process can be challenging, especially in patients with altered anatomy or a high body mass index (BMI), where visualization and access are limited [14]. One of the benefits of circular stapling is its ability to create a uniform and consistent anastomotic lumen, which may reduce the risk of postoperative stenosis [14]. A uniform lumen is particularly advantageous in maintaining consistent gastric emptying and reducing the variability in postoperative outcomes [15]. Additionally, some studies suggest that when performed properly, circular staplers may be more effective in preventing torsion or twisting at the anastomotic site, which can further minimize the risk of leaks and strictures [14,15].

Complication Rates

Circular stapling has a higher incidence of strictures compared to linear and hand-sewn techniques. Gonzalez et al. reported a stricture rate of 31% with circular stapling, significantly higher than the rates observed with hand-sewn (3%) and linear stapled techniques (0%) [10]. Additionally, Ramirez et al. observed a higher rate of ulcers (6.0%) in circular stapling, which could be due to increased tension at the anastomotic site [14].

However, circular stapling is associated with more consistent lumen diameters, which may reduce the risk of certain complications like stenosis. Despite this potential benefit, the higher overall complication rate, including leaks and ulcers, limits its widespread use, especially among less experienced surgeons [14].

Application in OAGB

The use of circular stapled anastomosis in OAGB is less common compared to linear stapling due to concerns about the higher incidence of strictures and the potential for bile reflux, which can exacerbate marginal ulcers [10]. The uniform anastomotic shape provided by circular staplers may be beneficial in controlling lumen size, but more research is needed to confirm its safety and efficacy in the context of OAGB.

Hand-sewn anastomosis

Hand-sewn anastomosis involves manually suturing the gastric pouch to the jejunal limb. This technique provides the greatest control over anastomotic size and tension, making it particularly useful in complex cases.

Technical Aspects

Hand-sewn anastomosis typically uses a two-layer approach: the first layer involves continuous absorbable sutures for mucosal approximation, while the second layer uses interrupted non-absorbable sutures for seromuscular closure [10]. This method allows for meticulous adjustments during surgery, accommodating individual patient anatomical variations and reducing the risk of complications such as leaks and strictures [10].

Operative Efficiency

Hand-sewn techniques have the longest operative times, averaging 215 minutes compared to 92.5 minutes for linear staplers and 110.6 minutes for circular staplers [10]. The extended duration is largely due to the intricate nature of the procedure, which requires careful suturing of both the mucosal and seromuscular layers to ensure a secure and leak-proof anastomosis.

The extended operative time can be a drawback, especially in high-volume centers where efficiency is paramount. Longer surgeries are associated with increased risks of anesthesia-related complications, such as respiratory or cardiovascular issues, and can contribute to longer hospital stays and recovery times [10]. This factor limits the widespread use of hand-sewn techniques in settings where rapid turnaround is necessary.

However, the precision and adaptability of the hand-sewn method offer significant advantages in complex cases. For example, in patients with previous abdominal surgeries or anatomical variations, where staplers may not provide adequate control, the hand-sewn technique allows for customized adjustments to accommodate unique anatomical challenges [16]. This flexibility is particularly valuable in revisional surgeries, where scar tissue and altered anatomy can complicate the creation of a new anastomosis.

Complication Rates

The hand-sewn technique has shown the lowest incidence of anastomotic complications among the three techniques. Gonzalez et al. reported a stricture rate of only 3%, compared to 31% in the circular stapled group and 0% in the linear stapled group [10]. Additionally, Maralani et al. reported an exceptionally low incidence of anastomotic leaks, with only one case out of 805 patients (0.1%), underscoring the safety and precision of the hand-sewn technique in OAGB [15].

Despite its advantages, the variability in outcomes is more pronounced with hand-sewn techniques, as they are heavily dependent on the surgeon's experience and skill level. Studies have shown that less experienced surgeons may encounter higher rates of complications such as leaks and strictures, underscoring the importance of specialized training and practice [15,16].

Application in OAGB

Hand-sewn anastomosis is particularly useful in OAGB, where precise control over the anastomotic size and tension is crucial to minimize bile reflux and other complications. This technique is recommended for complex cases or revisional surgeries where anatomical variations necessitate a tailored approach. However, its use is limited by the need for high surgical expertise and longer operative times, making it less practical in all clinical settings [16].

Discussion and implications for OAGB

Summary of Findings

Each anastomotic technique has unique benefits and drawbacks when applied to OAGB. Linear stapling offers shorter operative times and lower stricture rates but poses a higher risk of marginal ulcers. Circular stapling, while providing consistent lumen diameters, is associated with higher complication rates and requires significant surgical expertise. Hand-sewn techniques, though time-consuming, provide the best outcomes in terms of reducing strictures and leaks, making them ideal for complex cases.

Extrapolation to OAGB

While the literature on anastomotic techniques in OAGB is limited, findings from studies on RYGB and other bariatric procedures can be extrapolated to some extent. For example, the lower operative time and the low incidence of strictures in linear stapling suggest it may be a suitable option for OAGB, provided that the anastomotic diameter is carefully controlled to prevent marginal ulcers. Similarly, the precision offered by hand-sewn techniques can be beneficial in OAGB, particularly in preventing bile reflux, which is a common concern with this procedure.

Limitations in the Current Literature

There is a lack of large-scale, multicenter studies comparing these anastomotic techniques specifically in the context of OAGB. Most of the available data are extrapolated from RYGB and other bariatric surgeries, which may not fully reflect the unique challenges of OAGB. Future research should focus on direct comparisons of these techniques in OAGB to provide more definitive guidance for clinical practice. Studies should also investigate the long-term outcomes of different anastomotic techniques in OAGB, focusing on complication rates, weight loss outcomes, and quality of life. Randomized controlled trials and multicenter studies are needed to establish best practices and optimize surgical techniques for OAGB.

## Conclusions

Each anastomotic technique in OAGB has its own set of advantages and limitations. Linear stapling provides a balance of efficiency and safety, making it suitable for most standard cases. Circular stapling, while offering precise anastomotic control, requires greater surgical expertise and may be associated with higher complication rates. Hand-sewn anastomosis, though technically demanding, may offer good outcomes in reducing anastomotic complications, particularly in complex cases.

Future research should focus on large-scale, multicenter trials comparing these techniques to provide more definitive guidance on selecting the optimal anastomotic approach in OAGB. Additionally, innovations in surgical technology could potentially enhance the precision and efficiency of these techniques, improving outcomes in bariatric surgery.
